# Clinical outcomes and recurrence patterns in pancreatic ductal adenocarcinoma diagnosed at an early stage: insights from a multicenter cohort study in Japan

**DOI:** 10.1007/s00535-025-02340-x

**Published:** 2026-01-03

**Authors:** Juri Ikemoto, Yasutaka Ishii, Keiji Hanada, Tamito Sasaki, Yoshifumi Fujimoto, Atsushi Yamaguchi, Bunjiro Noma, Tomoyuki Minami, Masanobu Yukutake, Akihito Okazaki, Teruo Mouri, Shinya Nakamura, Kenichiro Uemura, Shinya Takahashi, Koji Arihiro, Shiro Oka

**Affiliations:** 1 Department of Gastroenterology, Graduate School of Biomedical and Health Sciences, Hiroshima University, 1-2-3 Kasumi, Minami-Ku, Hiroshima, 734-8551 Japan; 2 Department of Gastroenterology, Onomichi General Hospital, 1-10-23 Hirahara, Onomichi-Shi, Hiroshima, 722-8508 Japan; 3 Department of Gastroenterology, Hiroshima Prefectural Hospital, 1-5-54 Ujinakanda, Minami-Ku, Hiroshima-Shi, Hiroshima, 734-8530 Japan; 4 Department of Gastroenterology, Hiroshima General Hospital, 1-3-3 Jigozen, Hatsukaichi-Shi, Hiroshima, 738-8503 Japan; 5 Department of Gastroenterology, National Hospital Organization Kure Medical Center and Chugoku Cancer Center, 3-1, Aoyama-Cho, Kure-Shi, Hiroshima, 737-0023 Japan; 6 Department of Gastroenterology, Kure Kyosai Hospital, 2-3-28 Nishichuou, Kure-Shi, Hiroshima, 737-8505 Japan; 7 Department of Gastroenterology, Hiroshima Red Cross Hospital and Atomic-Bomb Survivors Hospital, 1-9-6 Sendamachi, Naka-Ku, Hiroshima-Shi, Hiroshima, 730-8619 Japan; 8 Department of Gastroenterology, Hiroshima City North Medical Center Asa Citizens Hospital, 2-1-1 Kabeminami, Asakita-Ku, Hiroshima-Shi, Hiroshima, 731-0293 Japan; 9 Department of Gastroenterology, National Hospital Organization Higashihiroshima Medical Center, Hiroshima, 513 Saijou-Cho Jike, Higashihiroshima-Shi, Hiroshima, 739-0041 Japan; 10 Department of Gastroenterology, Chugoku Rosai Hospital, 1-5-1 Hirotagaya, Kure-Shi, Hiroshima, 737-0193 Japan; 11 Department of Surgery, Graduate School of Biomedical and Health Sciences, Hiroshima University, 1-2-3 Kasumi, Minami-Ku, Hiroshima-Shi, Hiroshima, 734-8551 Japan; 12 Department of Anatomical Pathology, Hiroshima University Hospital, 1-2-3 Kasumi, Minami-Ku, Hiroshima-Shi, Hiroshima, 734-8551 Japan

**Keywords:** Pancreatic cancer, Early diagnosis of pancreatic cancer, Recurrence, Remnant pancreatic recurrence

## Abstract

**Background:**

The prognosis and recurrence patterns of early-diagnosed pancreatic ductal adenocarcinoma (PDAC), particularly following surgical resection, remain unclear.

**Methods:**

This multicenter retrospective study analyzed patients who underwent surgical resection for PDAC between 2005 and 2023. Patients were categorized according to pathological stages 0, I, and II. Recurrence patterns and survival outcomes were compared among the three groups. Multivariate analysis was performed to identify independent risk factors for remnant pancreatic recurrence, including early-stage disease, postoperative follow-up of more than 5 years, and receipt of adjuvant chemotherapy.

**Results:**

A total of 349 patients were included: 51 with stage 0, 77 with stage I, and 221 with stage II PDAC. The 5-year overall survival rates were 87%, 71%, and 49% for patients with stage 0, I, and II PDAC, respectively. Remnant pancreatic recurrence was observed in 10% of patients with stage 0 PDAC and 18% of patients with stage I PDAC, compared with 5% of those with stage II PDAC. Recurrence was significantly more frequent in stage I (*P* < 0.001) and tended to be higher in stage 0 (*P* = 0.062) than in stage II. Multivariate analysis identified pathological stage 0–I and postoperative follow-up of > 5 years as independent risk factors for remnant pancreatic recurrence.

**Conclusions:**

Patients with early-stage PDAC exhibit a higher risk of remnant pancreatic recurrence than those with stage II disease. These findings underscore the importance of long-term pancreas-focused surveillance in early-stage PDAC to enable timely detection of late recurrence and potentially improve patients outcomes.

**Supplementary Information:**

The online version contains supplementary material available at 10.1007/s00535-025-02340-x.

## Introduction

Patients with pancreatic ductal adenocarcinoma (PDAC) are associated with a poor prognosis, with a 5-year survival rate of less than 10% [1]. However, data from the Japan Pancreatic Cancer Registry indicated substantially better outcomes for early-stage cases, with 5-year survival rates of 85.8% for Union for International Cancer Control (UICC) stage 0 (in situ) and 80.4% for tumors less than 10 mm in diameter [2]. These findings indicate that even early-stage PDAC does not achieve a 5-year survival rate of 90%.

According to the same registry, individuals with UICC stage 0 and IA disease accounted for only 1.7% and 4.1% of the total patient population, respectively [2]. Despite the low frequency of early detection, advances in imaging and pathological diagnostic techniques have increased the likelihood of identify PDAC at an earlier stage [3–5].

Although early-stage PDAC is generally associated with a favorable prognosis, its recurrence patterns remain unclear. Recent studies suggest that recurrence within the remnant pancreas may be more common in early-stage PDAC, potentially due to multicenter carcinogenesis, residual pancreatic intraepithelial neoplasia (PanIN), or skip lesions [6–8]. These findings suggest that, although early-stage PDAC allows for prolonged survival, it may paradoxically be associated with a higher risk of remnant pancreatic recurrence, reflecting the extended observation period and the inherent biological characteristic of early lesions.

Based on this hypothesis, this multicenter study aimed to examine the recurrence patterns and survival outcomes in early-stage PDAC, with a particular focus on identifying clinical risk factors for recurrence within the remnant pancreas.

## Methods

### Patients

Patients who underwent pancreatic resection and were diagnosed with pathological stage 0 or I PDAC at Hiroshima University Hospital or its affiliated hospitals, and those diagnosed with pathological stage II PDAC following pancreatic resection at Hiroshima University Hospital between January 2005 and March 2023, were enrolled in this study. The study size was not predetermined by a sample size calculation; rather, all patients meeting the abovementioned eligibility criteria during the study period were enrolled.

The PDAC stage was determined histopathologically in accordance with the eighth edition of the General Rules for the Study of Pancreatic Cancer published by the Japan Pancreas Society (JPS) [9]. In this study, staging was performed based on the JPS criteria rather than the UICC classification, as stage I in the JPS system is defined not only by the tumor diameter but also by confinement within the pancreas. This approach enables a more precise evaluation of PDACs diagnosed at an earlier stage. Stage 0 corresponded to high-grade PanIN or pancreatic carcinoma in situ with no regional lymph node involvement or distant metastasis. Stage I indicated an invasive carcinoma confined to the pancreas without regional lymph node or distant metastasis. Stage IA corresponded to tumors with a maximum diameter of < 20 mm, whereas stage IB included tumors exceeding 20 mm. Stage IIA was defined by tumor extension beyond the pancreas, without invasion of the celiac or superior mesenteric artery and without regional lymph node or distant metastasis. Stage IIB included tumors that met the size and extent criteria for stages IA to IIA and were accompanied by regional lymph node metastasis, without evidence of distant metastasis. Stage III was defined by tumor invasion into the celiac and/or superior mesenteric artery, without evidence of distant metastasis. Stage IV was defined by the presence of distant metastasis, regardless of the primary tumor size or lymph node involvement.

Patients with stage IV PDAC were excluded due to the presence of distant metastases. Intraoperative peritoneal lavage cytology (CY) results were interpreted in accordance with the 8th edition of the JPS General Rules, in which a CY-positive finding is classified as distant metastasis; consequently, patients with CY-positive results were excluded. Patients with stage III PDAC were also excluded, as this stage is deemed unresectable and ineligible for curative surgery. Patients with intraductal papillary mucinous neoplasm (IPMN) concomitant with PDAC were included. However, those with high-grade dysplasia or IPMN-derived invasive cancer demonstrating a histological transition between IPMN and PDAC were excluded. Patients who received neoadjuvant chemotherapy (NAC) were prespecified for exclusion to maintain a uniform cohort undergoing upfront R0 resection for pathology-based, stage-wise comparisons. In our cohort, NAC was rarely administered to patients with stage 0/IA. Resection margin status was assessed in accordance with the 8th edition of the JPS General Rules [9], and international recommendations. R0 was defined as a minimum histological clearance of ≥ 1 mm from any margin, and R1 as a tumor at or within 1 mm. Only patients with R0 resection were included; those who had received NAC or had positive surgical margins were excluded.

Patients were followed every 2–3 months at the outpatient clinic of our hospital or an affiliated institution after initial surgery. PDAC recurrence was assessed using tumor markers, including serum carbohydrate antigen 19–9 (CA19-9) levels, and computed tomography (CT), magnetic resonance imaging (MRI), and positron emission tomography-CT (PET-CT). Remnant pancreatic cancer was defined as a tumor arising within the remnant pancreas, including the surgical margin, as detected on CT, MRI, and PET-CT. A representative patient with recurrence in the remnant pancreas is shown in Supplementary Fig. 1. Clinicopathological data including age, sex, tumor location, surgical procedure, pathological outcomes, adjuvant chemotherapy, development of extrapancreatic recurrence and recurrence in the remnant pancreas, prognosis, and date of last follow-up were collected from medical records. Follow-up data were collected until March 2024. None of the patients were lost to follow-up during the study period. As this retrospective study was conducted within a defined regional medical network, complete follow-up data were available from institutional records.

This study was conducted in accordance with the Declaration of Helsinki and approved by the ethics committee of Hiroshima University (approval number: E2024-007), which served as the lead institution. Ethics approval was also obtained from the respective ethics committees of all participating institutions. Owing to the retrospective nature of the study and the use of fully anonymized data, the requirement for informed consent was waived by the ethics committees.

### Outcomes

The primary endpoints were the recurrence rate and patterns of PDAC. In addition to evaluating the overall survival (OS) rate and recurrence patterns, patients were stratified into three groups based on the initial postoperative pathological stage: 0, I, and II. The secondary endpoints were the predictors of recurrence in the remnant pancreas. For the multivariate analysis, the following variables were included: time to recurrence, differences in prognosis according to recurrence patterns, age, sex, a preoperative CA19-9 level of > 37 U/mL, tumor location at initial surgery, surgical procedure, pathological stage, surgical margin status, adjuvant chemotherapy, and a follow-up period of > 5 years.

The survival endpoints and event definitions were as follows: OS was defined as death from any cause. Disease-specific survival (DSS) considered only PDAC-related deaths as events, censoring deaths from other causes (including perioperative or treatment-related mortality). Standard recurrence-free survival (RFS) was defined as the interval from surgery to either recurrence or death from any cause, whichever occurred first, with patients alive and recurrence-free censored at the last follow-up. Disease-specific RFS (DSRFS) was defined as the interval from surgery to the first documented recurrence, censoring deaths that occurred without prior recurrence. Lesions arising from other primary malignancies were not classified as PDAC recurrence.

### Statistical analysis

All statistical analyses were performed using JMP Pro 16.0.0 (SAS Institute Inc. Cary, North Carolina, USA). Continuous variables were compared using the Wilcoxon rank-sum test, and categorical variables were compared using the chi-square or Fisher’s exact test, as appropriate. OS and recurrence-free survival (RFS) rates were calculated using the Kaplan–Meier method, with differences assessed by the log-rank test. Univariate and multivariate analyses were performed using the Cox proportional hazards model to identify independent predictors of survival. A two-sided *P* value of < 0.05was considered significant. Missing data were minimal and unlikely to affect the results. Therefore, a complete case analysis was performed, without applying any imputation methods.

### Patient and public involvement

Patients and the public were not involved in the design, conduct, reporting, or dissemination of this research.

## Results

### Patient characteristics

After applying the predefined exclusion criteria, only 349 patients were included in the final analysis. All patients completed follow-up and were subsequently analyzed. Table 1 summarized the clinical characteristics of the 349 patients at the time of initial surgery. Tumors were located in the pancreatic head in 58% of patients and in the pancreatic body to tail in 42% of patients. Pathological staging was distributed as follows: stage 0, 51 patients (14%); stage IA, 61 patients (17%); stage IB, 16 patients (4%); stage IIA, 73 patients (21%); and stage IIB, 148 patients (42%). The median follow-up period after surgery was 54 months (interquartile range: 23–90 months).

**Table 1:** Clinicopathological characteristics of 349 patients who underwent resection for PDAC

Characteristics	Values
Age (years)	72 (65–78)
Sex (male/female)	182 (52)/167 (48)
Preoperative serum CA19-9 levels (U/mL)	33.3 (8.8–165)
IPMN on the preoperative images	77 (22)
EOB-MRI performed	40 (11)
Location of the tumor, head/body-tail	202 (58)/147 (42)
Surgical procedure, PD/DP/MP/TP	199 (57)/140 (40)/1 (0.3)/9 (3)
Pathological stage, 0/IA/IB/IIA/IIB	51 (15)/61 (17)/16 (5)/73 (21)/148 (42)
Histological type, CIS/wel/mod/por/others	51 (15)/111 (32)/156 (45)/20 (6)/11 (3)
Presence of intraductal lesions in the surgical specimen	207/323 (64)
Receipt of adjuvant chemotherapy	240 (69)
Adjuvant chemotherapy regimen: G/S/GS/others	14 (4)/55 (16)/169 (48)/2 (0.6)
Completion of adjuvant chemotherapy	219 (63)
Follow-up period after surgery (months)	54 (23–90)

Data are expressed as numbers (percentages) or median values (interquartile ranges)

Abbreviations: PDAC, pancreatic ductal adenocarcinoma; CA19-9, carbohydrate antigen 19–9; IPMN, intraductal papillary mucinous neoplasm; EOB-MRI, Gadoxetic acid-enhanced magnetic resonance imaging; PD, pancreaticoduodenectomy; DP, distal pancreatectomy; MP, middle pancreatectomy; TP, total pancreatectomy; CIS, carcinoma in situ; wel, well differentiated adenocarcinoma; mod, moderate differentiated adenocarcinoma; por, poorly differentiated adenocarcinoma; G, gemcitabine; S, S-1; GS, gemcitabine plus S-1

### Comparison of clinicopathological factors by pathological stages

**Fig. 1:**
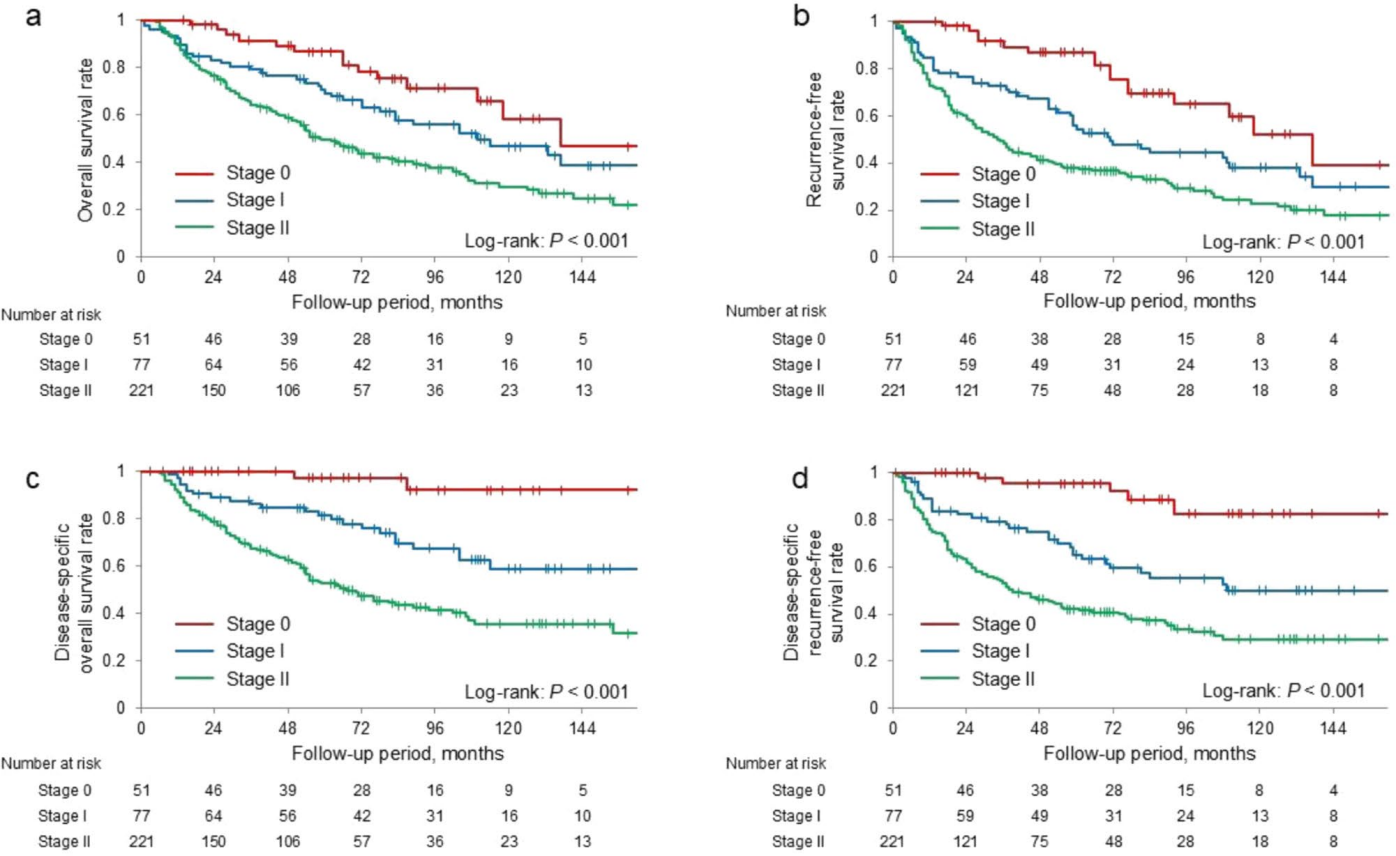
Kaplan–Meier analysis of the overall survival and recurrence-free survival stratified by pathological stage. **a** Overall survival, **b** recurrence-free survival, **c** disease-specific overall survival rate, and **d** disease-specific recurrence-free survival were significantly higher in patients with 0 and I pancreatic ductal adenocarcinoma than in those with stage II (log-rank test, all *P* < 0.001)

Table 2 presents the comparison of clinicopathological factors among patients with stages 0, I, and II. Tumor location differed significantly among the three groups (*P* < 0.001), with tumors more frequently located in the pancreatic body/tail in stages 0 and I than compared with stage II (*P* < 0.001 for both comparisons). Consistent with this distribution, distal pancreatectomy was performed more often in patients with stages 0 and I than in patients with stage II (*P* < 0.001 for both comparisons). Patients with stage II disease were significantly more likely to receive adjuvant chemotherapy than those with stage 0 and I disease (*P* < 0.001 for all comparisons). Additionally, patients with stage II disease demonstrated significantly larger tumor sizes and poorer histological differentiation than those with stage I disease (*P* < 0.001 and *P* = 0.026, respectively). Recurrence rates increased progressively with stage: stage I > stage 0 (*P* < 0.001) and stage II > stage I (*P* = 0.011). The median follow-up period was significantly shorter for patients with stage II disease than for those with stage 0 and I (*P* < 0.001 for both comparisons). The 5-year OS rates were 87%, 71%, and 49%, whereas the 10-year OS rates were 58%, 47%, and 30% for stages 0, I, and II, respectively. The corresponding 5-year RFS rates were 87%, 53%, and 38%, whereas the 10-year RFS rates were 52%, 38%, and 23%, respectively. The corresponding 5-year DSS rates were 97%, 81%, and 52%, whereas the 10-year DSS rates were 92%, 59%, and 35%, respectively. The corresponding 5-year DSRFS rates were 95%, 65%, and 33%, whereas the 10-year DSRFS rates were 82%, 50%, and 29%, respectively. The OS, RFS, DSS and DSRFS rates of stage 0 and I patients were significantly higher than those of stage II patients (log-rank test, *P* < 0.001 for all comparisons) (Fig. 1).

**Table 2:** Comparison of clinicopathological characteristics among the three groups classified by pathological stage

Characteristics	Stage 0 (n = 51)	Stage I (n = 77)	Stage II (n = 221)	*P* value
Age (years)	73 (66–78)	72 (64–78)	72 (66–78)	0.529
Sex (male/female)	30 (59)/21 (41)	37 (48)/40 (52)	115 (52)/106 (48)	0.489
Preoperative CA19-9 level (U/mL)	8 (3–14)	23 (8–69)	79 (16–344)	< 0.001
IPMN on the preoperative images	18 (35)	19 (25)	40 (18)	0.023
EOB-MRI performed	2 (4)	5 (6)	33 (15)	0.033
Location of the tumor: head/body-tail	14 (28)/37 (73)	34 (44)/43 (56)	154 (70)/67 (30)	< 0.001
Intraoperative peritoneal lavage cytology	25 (49)	45 (58)	221 (100)	< 0.001
Tumor diameter (mm)	–	15 (5–20)	28 (20–35)	< 0.001
Surgical procedure: PD/DP/TP	14 (27)/35 (69)/2 (4)	32 (42)/40 (52)/4 (5)	153 (69)/65 (29)/3 (1)	< 0.001
Histologic differentiation: wel/mod·por	–	37 (48)/31 (40)	74 (34)/145 (66)	0.002
Presence of low-grade PanIN in the surgical specimen	18/43 (42)	32/64 (50)	157/216 (73)	< 0.001
Receipt of adjuvant chemotherapy	1 (2)	48 (62)	191 (86)	< 0.001
Regimen of chemotherapy: G/S/GS	0/1 (2)/0	10 (13)/29 (38)/7 (9)	4 (2)/25 (11)/162 (73)	< 0.001
Completion of adjuvant chemotherapy	1 (2)	40 (52)	178 (81)	< 0.001
Recurrence	5 (10)	31 (40)	126 (57)	< 0.001
Follow-up period after surgery (months)	77 (48–110)	76 (38–111)	43 (19–73)	< 0.001

Data are expressed as numbers (percentages) or median values (interquartile ranges)

Abbreviations: CA19-9, carbohydrate antigen 19–9; IPMN, intraductal papillary mucinous neoplasm; EOB-MRI, Gadoxetic acid-enhanced magnetic resonance imaging; PD, pancreaticoduodenectomy; DP, distal pancreatectomy; TP, total pancreatectomy; wel, well; mod, moderate; por, poor; PanIN, pancreatic intraepithelial neoplasia; G, gemcitabine; S, S-1; GS, gemcitabine plus S-1

### Comparison of recurrence patterns by pathological stage

Table 3 presents the recurrence patterns of patients with stages 0, I, and II disease. Liver metastasis was significantly more common in stage II than in stage 0 (*P* < 0.001) and stage I (*P* = 0.031), with no significant difference between stages 0 and I (*P* = 0.156). Local recurrence was also more common in stage II than in stage 0 (*P* = 0.029) or stage I (*P* = 0.031); however, no significant differences were found between stages 0 and I (*P* = 1.000). Similarly, lung metastasis occurred more frequently in stage II than in stage 0 (*P* < 0.001) or stage I (*P* = 0.046), whereas stages 0 and I did not differ significantly (*P* = 0.156). Conversely, remnant pancreatic recurrence was more common in stages 0 and I than in stage II (*P* = 0.062 and *P* < 0.001, respectively). However, no significant difference was found between stages 0 and I (*P* = 0.216).

**Table 3:** Comparison of recurrence patterns among the three groups stratified by pathological stage

	Stage 0 (n = 51)	Stage I (n = 77)	Stage II (n = 221)	*P* value
Recurrence	5 (10)	31 (40)	126 (57)	< 0.001
Liver	0 (0)	5 (6)	36 (16)	0.001
Local	0 (0)	1 (1)	19 (9)	0.010
Lung	0 (0)	5 (6)	34 (15)	0.002
Lymph	0 (0)	0 (0)	12 (5)	0.027
Peritoneum	0 (0)	3 (4)	13 (6)	0.184
Remnant pancreas	5 (10)	14 (18)	8 (4)	< 0.001
Others	0 (0)	3 (4)	4 (2)	0.288

Data are expressed as numbers (percentages)

### Recurrence within the remnant pancreas

**Fig. 2:**
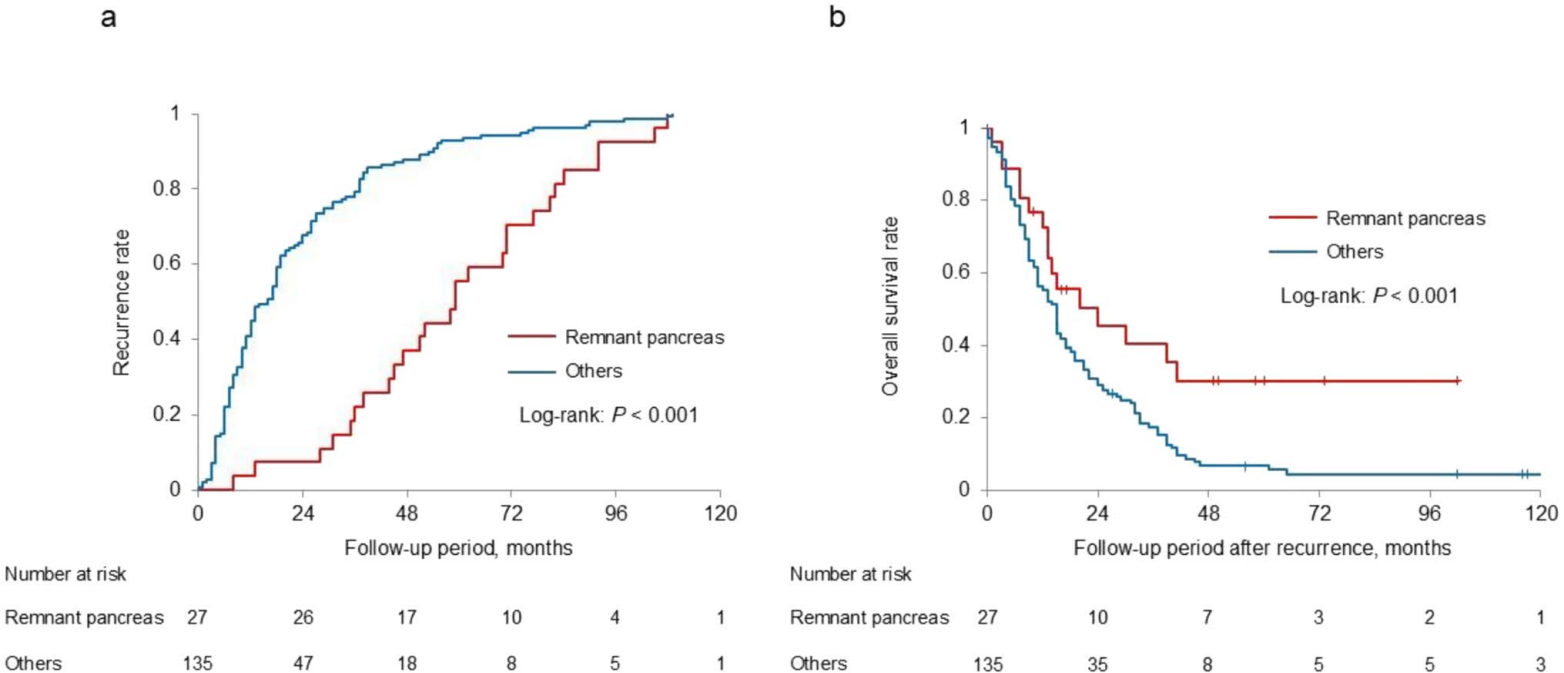
Comparison of recurrence between the remnant pancreas and other sites. **a** The median recurrence-free survival time was significantly longer in patients with recurrence within the remnant pancreas than in those with recurrence at other sites (median survival time: 51 vs. 10 months, *P* < 0.001). **b** Kaplan–Meier analysis of the overall survival demonstrated significantly better survival in patients with recurrence within the remnant pancreas compared with those who had recurrence at other sites (median survival time: 20 vs. 14 months, *P* = 0.015)

Table 4 summarizes the results of the univariate and multivariate analyses of risk factors associated with recurrence within the remnant pancreas. In the univariate analysis pathological stages 0 and I, receipt adjuvant chemotherapy, and a follow-up period of > 5 years were identified as significant factors (*P* < 0.001, *P* = 0.048 and *P* = 0.001, respectively). Multivariate analysis revealed that pathological stages 0 or I, along with a follow-up period of > 5 years, were independent risk factors for recurrence within the remnant pancreas (odds ratio [OR]: 3.757, 95% confidence interval [CI]: 1.404–10.502, *P* = 0.008; OR: 3.295, 95% CI: 1.255–9.767, *P* = 0.015). The Kaplan–Meier analysis of RFS, comparing recurrence within the remnant pancreas and recurrence at other sites, is presented in Fig. 2. The median RFS duration was significantly longer among those with recurrence within the remnant pancreas than among those with recurrence at other sites (median survival time: 51 vs. 10 months, *P* < 0.001). Conversely, there were no significant differences in the timing of either remnant pancreatic recurrence or recurrence at other sites among stages (log-rank tests, *P* = 0.138 and *P* = 0.215, respectively; Supplementary Fig. 2). Moreover, in the disease-specific analysis, in which distant metastasis, local recurrence, and deaths from causes unrelated to PDAC were censored and only remnant pancreatic recurrence was treated as the event, the Kaplan–Meier curves for stages 0, I, and II were nearly identical (Supplementary Fig. 3). Notably, the disease-specific recurrence-free survival curve for stage II was significantly higher than that for stage I (*P* = 0.018). Table 5 summarizes the clinical characteristics of patients who experienced recurrence within the remnant pancreas. Treatment included remnant pancreatectomy (*n* = 15), chemotherapy (*n* = 7), and best supportive care (*n* = 5). Among those who underwent remnant pancreatectomy, 10 patients survived for at least 2 years following the second surgery. The Kaplan–Meier analysis of OS after recurrence, comparing patients with recurrence within the remnant pancreas with those with at other sites, is presented in Fig. 2. By contrast, OS after recurrence was significantly longer among patients with recurrence within the remnant pancreas compared with those who experienced recurrence at other sites (median survival time: 20 vs. 14 months, *P* = 0.015).

**Table 4:** Univariate and multivariate analyses of risk factors for recurrence in the remnant pancreas

	Recurrence	No recurrence	Univariate analysis		Multivariate analysis	
Parameters	(n = 27)	(n = 322)	Odds ratio (95% CI)	*P* value	Odds ratio (95% CI)	*P* value
Age > 70 years	18	198	0.798 (0.348–1.832)	0.595		
Sex (male)	13	169	1.189 (0.542–2.610)	0.665		
Preoperative CA19-9 level > 37 U/mL	12	156	1.175 (0.533–2.588)	0.689		
IPMN on the preoperative images	7	70	0.794 (0.323–1.953)	0.614		
Location of the tumor: head	13	189	1.530 (0.697–3.361)	0.286		
Surgical procedure: PD	12	187	1.855 (0.840–4.096)	0.154		
Presence of low-grade PanIN in the surgical specimen	14	193	0.769 (0.330–1.791)	0.541		
pStage: 0–I	19	109	5.397(2.199–13.247)	< 0.001	3.757 (1.404–10.502)	0.008
Receipt of adjuvant chemotherapy	14	226	0.457 (0.207–1.010)	0.048	1.154 (0.453–2.981)	0.764
Completion of adjuvant chemotherapy	14	205	0.615 (0.279–1.352	0.223		
Follow-up period: ≥ 5 years	20	138	3.810 (1.567–9.263)	0.001	3.295 (1.255–9.767)	0.015

Abbreviations: CA19-9, carbohydrate antigen 19–9; IPMN, intraductal papillary mucinous neoplasm; PD, pancreaticoduodenectomy; PanIN, pancreatic intraepithelial neoplasia; CI, confidence interval

**Table 5:** Clinical characteristics of patients with recurrence in the remnant pancreas

Initial surgery						Recurrence			
Age, sex	Stage	CA19-9 (U/mL)	Tumor size (mm)	Surgical procedure	Adjuvant CT	Interval (m)	Therapy	Stage	Prognosis (after initial surgery, m)
65, F	0	2	Tis	DP	ND	36	RP	IIA	Dead (60)
70, M	0	44	Tis	PD	ND	92	RP	IIA	Alive (165)
70, M	0	8	Tis	DP	ND	77	RP	IIB	Dead (87)
73, M	0	15	Tis	DP	ND	71	CT	IV	Alive (78)
78, M	0	9	Tis	DP	ND	28	BSC	IA	Dead/UD (32)
60, M	IA	97	1	DP	G	38	RP	IIA	Dead (78)
69, F	IA	61	1	DP	S	51	BSC	IV	Dead (54)
77, F	IA	24	1	DP	ND	71	BSC	IIA	Dead (83)
82, F	IA	2.4	5	DP	ND	59	CT	IB	Alive (76)
72, F	IA	7.8	6	DP	S	59	RP	IB	Alive (108)
64, M	IA	–	10	PD	GS	13	RP	III	Dead (29)
71, F	IA	4	10	PD	G	31	CT	IIA	Dead (62)
74, F	IA	8	10	DP	ND	44	RP	IA	Alive (147)
79, F	IA	22	10	DP	ND	84	RP	IIB	Dead (104)
71, F	IA	55	15	DP	G	58	CT	IV	Dead (72)
76, M	IA	130	15	DP	ND	81	CT	IV	Dead (89)
39, M	IA	2	20	PD	ND	62	RP	III	Dead (104)
79, M	IB	68.8	21	DP	S	70	BSC	III	Dead (83)
79, F	IB	38.5	40	PD	ND	8	CT	III	Dead (9)
70, F	IIA	163	22	PD	ND	82	RP	IIB	Dead (107)
81, F	IIA	8	25	PD	GS	35	CT	IIA	Alive (52)
84, M	IIA	295	30	PD	GS	92	RP	IIA	Alive (153)
69, M	IIA	12	45	PD	GS	105	BSC	IV	Dead (109)
53, F	IIB	56	13	DP	GS	108	RP	IIB	Alive (159)
66, M	IIB	20	25	PD	GS	52	RP	IIA	Dead/UD (62)
68, M	IIB	91	30	PD	GS	45	RP	IIA	Alive (103)
71, M	IIB	748	32	PD	GS	47	RP	IIB	Dead (54)

Abbreviations: y, years; CA19-9, carbohydrate antigen; m, months; CT, chemotherapy; PD, pancreaticoduodenectomy; DP, distal pancreatectomy; CT, chemotherapy; ND, not done; GS, gemcitabine plus S-1; G, gemcitabine; S, S-1; RP, remnant pancreatectomy; UD, unrelated disease; BSC, best supportive care

## Discussion

In this multicenter cohort study, we examined the prognosis and patterns of recurrence in resected PDAC. The 5-year OS rates for stage 0, I, and II were 87%, 71%, and 49%, respectively, and OS in patients with stage 0 and I disease was significantly higher than that in stage II patients. The recurrence rates by stage were 10% in stage 0, 40% in stage I, and 57% in stage II, indicating that earlier-stage diagnosis was associated with a significantly lower overall risk of recurrence. Recurrence patterns also varied by stage: both stage 0 and stage I showed a high proportion of remnant pancreatic recurrence (100% and 45%, respectively), whereas this pattern was uncommon in stage II (6%). Thus, the proportion of remnant pancreatic recurrence was significantly higher in patients diagnosed at an earlier stage. Multivariable analysis identified stage 0–I and a postoperative follow-up period longer than 5 years as independent predictors of remnant pancreatic recurrence. Furthermore, in the disease-specific analysis that censored distant metastasis, local recurrence, and deaths from causes unrelated to PDAC and treated only remnant pancreatic recurrence as the event, the Kaplan–Meier curves were nearly identical across stages. This finding supports the hypothesis that the apparent enrichment of remnant pancreatic recurrence in early-stage PDAC is largely attributable to longer survival and extended surveillance, rather than an intrinsic, stage-dependent biological predisposition.

Several reports have documented the widespread intraductal extension of high-grade PanIN [10, 11]. Aimoto et al. [10] reported a patient who underwent distal pancreatectomy for high-grade PanIN, followed by completion pancreatectomy that revealed extensive and multicentric high-grade PanIN. Ito et al. [11] described a similar patient with multifocal high-grade PanIN interspersed with low-grade lesions. In the present cohort, one patient with stage 0 PDAC underwent total pancreatectomy, and high-grade PanIN was identified in the pancreatic head, demonstrating continuous intraductal extension to the distal duct [12]. Given that some PanIN lesions demonstrate intraductal spread and skip areas, postoperative surveillance should account for the possibility of residual or undetected lesions in the remnant pancreas, beyond the findings of intraoperative margin assessment. Yachida et al. [13] estimated that the progression from normal ductal epithelium to non-invasive carcinoma takes approximately 11.7 years, with an additional 6.8 years required for progression to invasive carcinoma and metastasis. This prolonged natural history suggests that in cases of remnant pancreatic recurrence observed in the present study, high- or low-grade PanIN may have already been present in the remnant pancreas at the time of initial surgery and gradually progressed to invasive cancer over several years. Therefore, long-term pancreas-focused surveillance is essential, particularly in patients with early-stage PDAC, who achieve prolonged survival.

However, patients diagnosed with stage II PDAC at the time of initial surgery also achieved long-term survival, with some developing recurrence within the remnant pancreas as late as 92 months postoperatively. Furthermore, a follow-up period exceeding 5 years independently predicted remnant pancreatic recurrence, regardless of disease stage. The lower incidence of such recurrence in patients with advanced-stage disease, as reported in previous studies [6, 14] and corroborated by our findings, is therefore likely explained by limited long-term survival and competing risks as many of these patients die of liver metastases or peritoneal dissemination before heterotopic and metachronous recurrence in the remnant pancreas can become clinically apparent. In the disease-specific analysis focusing on remnant pancreatic recurrence, in which distant metastasis, local recurrence, and deaths from other causes were censored and only remnant pancreatic recurrence was treated as the event, the Kaplan–Meier curves for stages 0, I, and II were nearly identical, with the DSRFS curve for stage II even slightly higher than that for stage I. These observations suggest that, in the absence of distant metastasis or local recurrence and with sufficiently prolonged survival, recurrence within the remnant pancreas becomes more likely to be detected. Consistent with this interpretation, no significant stage-related differences were observed in the timing of either remnant pancreatic recurrence or recurrence at other sites. A prospective registry of patients undergoing pancreatectomy demonstrated that S-1 adjuvant chemotherapy achieved a 5-year OS of 44.1% and a median survival of 46.5 months [15]. These findings established adjuvant chemotherapy as the standard of care following PDAC resection. In the present study, adjuvant chemotherapy was not associated with the risk of remnant pancreatic recurrence. However, among patients with stage II PDAC, those who received adjuvant chemotherapy exhibited significantly longer survival than those who did not (median survival time: 73 months vs. 25 months; log-rank *P* < 0.001). Recent studies have further demonstrated the efficacy of regimens such as modified FOLFIRINOX, gemcitabine plus capecitabine, and gemcitabine plus nab-paclitaxel [16–18]. With the availability of more effective therapeutic options, even patients with advanced-stage PDAC may achieve long-term survival, which could increase the likelihood of detecting recurrence within the remnant pancreas.

The distinction between whether recurrence within the remnant pancreas represents regrowth of the initial tumor or a metachronous, second primary carcinoma remains a significant clinical and biological concern. Lichini et al. [19] investigated six patients who developed PDAC in the remnant pancreas following primary tumor resection. Through histopathological comparison and next-generation sequencing targeting 11 driver genes (*KRAS, TP53, SMAD4, CDKN2A, GNAS, RNF43, TGFBR2*, *ARID1A*, *BRAF*, *MAP2K4*, and *PIK3CA*), three patients were identified as having conventional recurrences with identical morphology and shared mutations, two patients had second primaries with distinct profiles, and one was inconclusive. Their study further noted that positive surgical margins at the initial resection corresponded to conventional recurrences, whereas negative margins and survival beyond 4 years were associated with different mutations, suggesting metachronous recurrence. Similarly, Hashimoto et al. [20] conducted a multicenter study involving 50 patients with PDAC in the remnant pancreas. Among 17 patients who underwent RAS mutation analysis, only two exhibited matching *KRAS* or *HRAS* mutations between the initial and recurrent tumors. Most patients displayed unique mutational profiles, supporting the hypothesis that many of these lesions were metachronous and heterotopic in origin. In the present study, tissue or genetic analyses were not performed at the time of initial surgery or recurrence. However, all patients had negative surgical margins at the initial surgery, and many had a postoperative follow-up period exceeding 3 years. Based on these observations and prior evidence, it is plausible that recurrence within the remnant pancreas in our cohort represents metachronous and heterotopic recurrence rather than regrowth of the original tumor. To further explore this possibility, molecular and genetic analyses of paired primary and recurrent tumor samples are planned to elucidate clonal relationships and inform improved surveillance strategies.

Patients with remnant pancreatic recurrence appeared to have better prognosis compared with those who experienced other types of recurrence. Notably, approximately half of these patients underwent total remnant pancreatic resection, and some survived for more than 10 years after the initial surgery. These observations may suggest the potential importance of diagnosing remnant pancreatic cancer while it is still resectable. Suzuki et al. [6] reported that resection of the remnant pancreatic recurrence was feasible in about half of PDAC cases and led to favorable outcomes. The National Comprehensive Cancer Network guidelines recommend surveillance with serum CA19-9 and CT every 3 to 6 months for 2 years after pancreatectomy [21]. Several reports also suggest that endoscopic ultrasonography (EUS) has superior detectability to CT and MRI for small PDAC [4, 22]. CT alone may miss small lesions in the remnant pancreas, making EUS essential for follow-up. Maruyama et al. [23] reported that even after pancreatic surgery, EUS achieved high visualization rates of the remnant pancreas. Therefore, for PDAC, clinicians should consider a follow-up protocol incorporating CT and EUS focused on the remnant pancreas, especially for early–stage PDAC cases with a favorable long-term prognosis.

Nonetheless, in our cohort, a subset of patients showed rising serum CA19-9 levels before radiologic confirmation of recurrence and experienced rapid disease progression, in some cases proceeding directly to best supportive care. These early disease-specific deaths, which occurred despite regular surveillance with tumor markers and cross-sectional imaging, are more consistent with aggressive tumor biology than with delayed or insufficient follow-up. In addition, advanced age and postoperative functional decline may have limited post-recurrence treatment options in some patients and further contributed to early mortality.

In clinical practice, surveillance for cancers such as gastric, colorectal, and lung cancer is often discontinued after 5 years of follow-up [21]. However, in the present study, remnant pancreatic recurrence was observed even beyond 5 years postoperatively, without evidence of a plateau. This finding highlights the necessity for extended follow-up in patients with PDAC. Although specific risk factors for remnant pancreatic recurrence (such as smoking, alcohol consumption, or chronic pancreatitis) were not identified in this study, the results support the need for prolonged follow-up focused on the remnant pancreas, particularly in patients with early-stage PDAC. Current guidelines recommend regular endoscopic surveillance beyond 5 years after endoscopic treatment for gastric cancer, combined with *Helicobacter pylori* eradication [24], to prevent and enable early detection of metachronous recurrence. Similarly, for PDAC, clinicians should consider implementing structured, long-term surveillance protocols targeting the remnant pancreas, even beyond the 5-year postoperative period, to facilitate early detection of late recurrence and improve patient outcomes.

This study has some limitations. First, its retrospective design and the inclusion of patients with stage II disease from a single institution may have introduced selection bias. Second, to maintain consistency in initial management, patients who received NAC were excluded, and only those who underwent R0 resection were included, which may limit the generalizability of the findings. Future studies incorporating patients treated with contemporary neoadjuvant regimens will be important to determine whether our findings regarding remnant pancreatic recurrence also apply to NAC-treated cohorts. Third, pathological comparisons between the primary tumor and the recurrence within the remnant pancreas were not performed, making it difficult to determine true multicentric carcinogenesis from local recurrence. Fourth, the study period spanned 18 years (2005–2023), during which imaging modalities and diagnostic accuracy for detecting small remnant pancreatic lesions likely improved over time. Although a standardized surveillance protocol, primarily consisting of contrast-enhanced CT every 3–6 months and supplemented with MRI or EUS as indicated, was employed across centers, temporal advances in imaging technology may have enhanced the sensitivity for detecting small or subclinical remnant recurrences in recent years, potentially affecting detection rates.

Although the present study demonstrated that early-stage PDAC was associated with a higher incidence of recurrence within the remnant pancreas, these findings should be interpreted with caution due to the retrospective design and potential selection bias. Although the results are consistent with those of previous single-center studies, the multicenter nature of this analysis provides stronger supporting evidence. The higher recurrence rate observed in patients with early-stage PDAC may be attributable to longer follow-up durations and the potential for latent multicentric carcinogenesis. Further prospective studies are warranted to validate these findings and establish optimized surveillance strategies for patients with early-stage PDAC.

Because this study was conducted within a limited geographical region of Japan, caution should be exercised when generalizing the findings to broader populations. However, the inclusion of multiple affiliated institutions and the application of standardized diagnostic and surgical procedures enhance the external validity and reliability of the results.

In conclusion, early-stage PDAC is associated with prolonged survival but also demonstrates a higher incidence of recurrence within the remnant pancreas. Close and long-term monitoring of the remnant pancreas in patients who survive beyond 5 years after resection may contribute to further improvements in prognosis.

## Supplementary Information

Below is the link to the electronic supplementary material.

Supplementary file1 (PDF 535 KB)
