# Supplementary material for: Clinical outcomes and recurrence patterns in pancreatic ductal adenocarcinoma diagnosed at an early stage: insights from a multicenter cohort study in Japan
Source: J Gastroenterol. 2026 Jan 3;61(3):334–44. doi: 10.1007/s00535-025-02340-x (PMC12987814; doi:10.1007/s00535-025-02340-x)
Supplement: Supplementary file 1 — Supplementary file1 (PDF 535 KB) [file 535_2025_2340_MOESM1_ESM.pdf]

Clinical outcomes and recurrence patterns in pancreatic ductal adenocarcinoma diagnosed at an early stage: Insights from a multicenter cohort study in Japan

Juri Ikemoto,[1] Yasutaka Ishii,[1] Keiji Hanada,[2] Tamito Sasaki,[3] Yoshifumi Fujimoto,[4] Atsushi Yamaguchi,[5] Bunjiro Noma,[6] Tomoyuki Minami,[7] Masanobu Yukutake,[8] Akihito Okazaki,[9] Teruo Mouri,[10] Shinya Nakamura,[1] Kenichiro Uemura,[11] Shinya Takahashi,[11] Koji Arihiro,[12] and Shiro Oka[1]

[1] Department of Gastroenterology, Graduate School of Biomedical & Health Sciences, Hiroshima University, 1-2-3 Kasumi, Minami-ku, Hiroshima, 734-8551, Japan

[2] Department of Gastroenterology, Onomichi General Hospital, 1-10-23 Hirahara, Onomichi-shi, Hiroshima 722-8508, Japan

[3] Department of Gastroenterology, Hiroshima Prefectural Hospital, 1-5-54 Ujinakanda, Minami-ku, Hiroshima-shi, Hiroshima 734-8530, Japan

[4] Department of Gastroenterology, Hiroshima General Hospital, 1-3-3 Jigozen, Hatsukaichi-shi, Hiroshima 738-8503, Japan

[5] Department of Gastroenterology, National Hospital Organization Kure Medical Center and Chugoku Cancer Center, 3-1, Aoyama-cho, Kure-shi, Hiroshima 737-0023, Japan

[6] Department of Gastroenterology, Kure Kyosai Hospital, 2-3-28 Nishichuou, Kure-shi, Hiroshima 737-8505, Japan

[7] Department of Gastroenterology, Hiroshima Red Cross Hospital & Atomic-bomb Survivors Hospital, 1-9-6 Sendamachi, Naka-ku, Hiroshima-shi, Hiroshima 730-8619, Japan

[8] Department of Gastroenterology, Hiroshima City North Medical Center Asa Citizens Hospital, 2-1-1 Kabeminami, Asakita-ku, Hiroshima-shi, Hiroshima 731-0293, Japan

[9] Department of Gastroenterology, National Hospital Organization Higashihiroshima Medical Center, Hiroshima, 513 Saijou-cho Jike, Higashihiroshima-shi, Hiroshima 739-0041, Japan

[10] Department of Gastroenterology, Chugoku Rosai Hospital, 1-5-1 Hirotagaya, Kure-shi, Hiroshima 737-0193, Japan

[11] Department of Surgery, Graduate School of Biomedical & Health Sciences, Hiroshima University, 1-2-3 Kasumi, Minami-ku, Hiroshima-shi, Hiroshima 734-8551, Japan

[12] Department of Anatomical Pathology, Hiroshima University Hospital, 1-2-3 Kasumi, Minami-ku, Hiroshima-shi, Hiroshima 734-8551, Japan

Corresponding author:

Yasutaka Ishii

Department of Gastroenterology, Graduate School of Biomedical & Health Sciences,

Hiroshima University, 1-2-3 Kasumi, Minami-ku, Hiroshima, 734-8551, Japan

Phone number: +81-82-257-5193

Fax number: +81-82-257-5194

E-mail: yishii@hiroshima-u.ac.jp

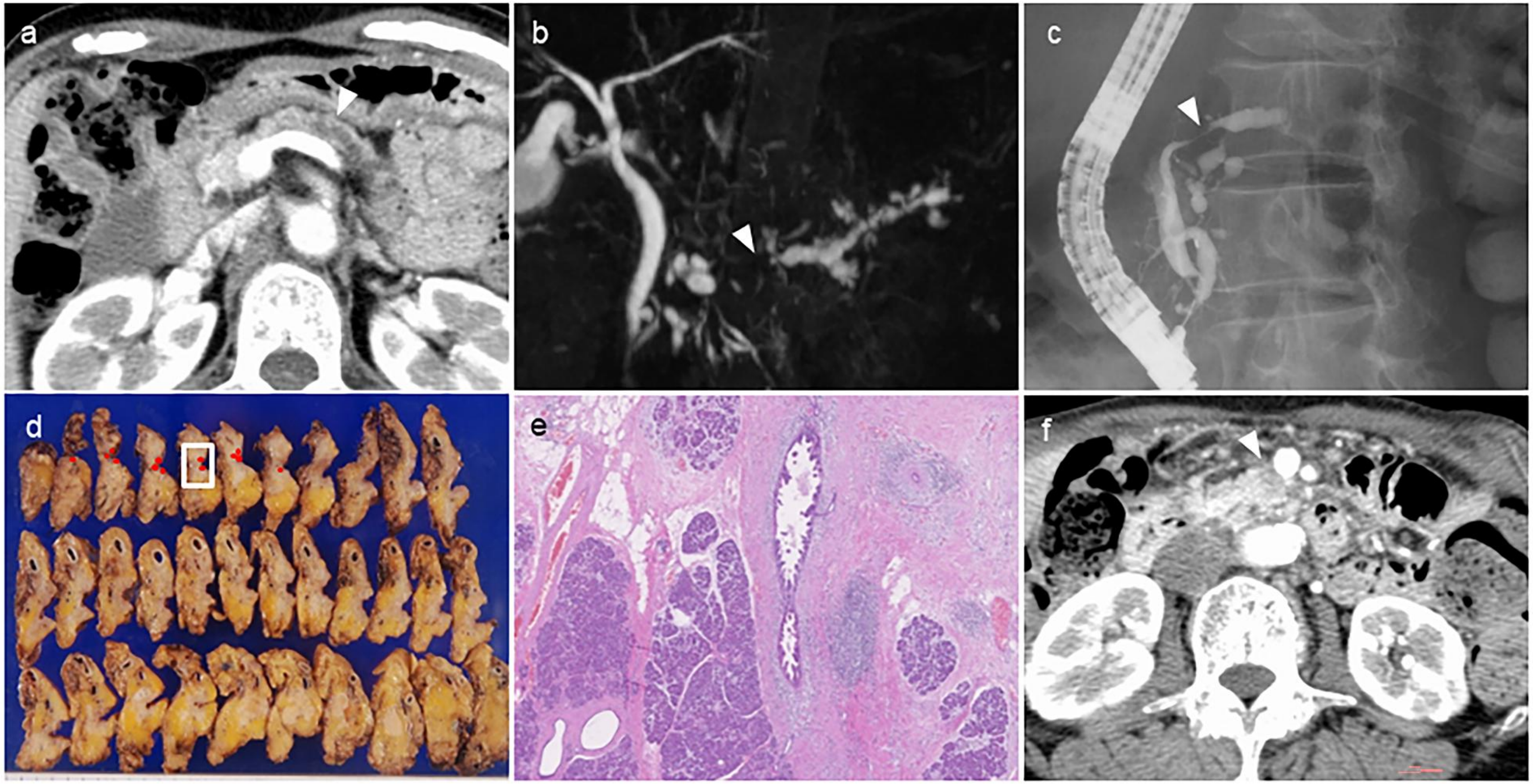
a
b
c
d
e
f

Supplementary Fig. 1. A representative case of early-stage pancreatic ductal adenocarcinoma that recurred in the remnant pancreas. (a) Contrast-enhanced computed tomography (CT) shows dilatation of the main pancreatic duct (MPD) from the body to the tail of the pancreas without an obvious mass lesion (arrowhead). (b) Magnetic resonance cholangiopancreatography reveals stenosis of the MPD in the pancreatic body (arrowhead) with upstream ductal dilatation. (c) Endoscopic retrograde pancreatography demonstrates stenosis of the MPD in the pancreatic body (arrowhead). (d) Resected specimen of distal pancreatectomy showing high-grade pancreatic intraepithelial neoplasia (PanIN) and microinvasion in the pancreatic body (red dots). (e) Histopathological examination of the boxed area in panel d reveals high-grade PanIN. (f) CT obtained four years after the initial surgery shows a low-density mass in the remnant pancreas.

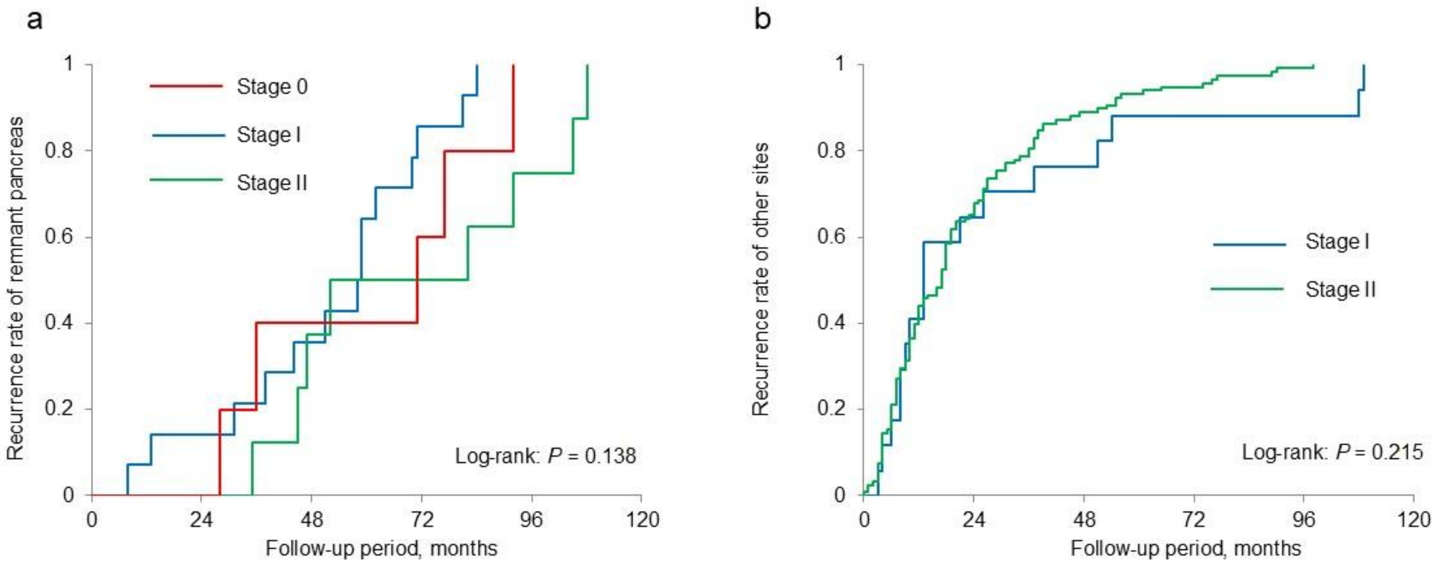

Number at risk						
Stage 0	5	5	4	3	0	0
Stage I	14	13	10	3	0	0
Stage II	8	8	7	5	3	0

Number at risk						
Stage I	17	7	5	3	2	0
Stage II	118	41	14	6	2	0

Supplementary Fig. 2. Comparison of the time to recurrence in the remnant pancreas and at other sites according to disease stage.

(a) Median recurrence-free survival for patients with isolated remnant pancreatic recurrence was not significantly different among stages (log rank test, P = 0.138).

(b) Median recurrence-free survival for patients with isolated recurrence at other sites was not significantly different among stages (log rank test, P = 0.215).

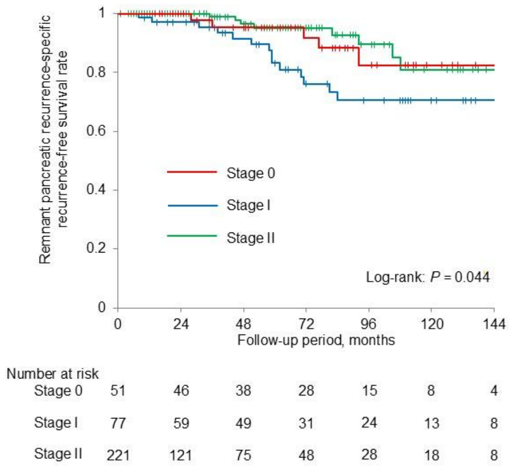

Supplementary Fig. 3. Remnant pancreatic recurrence-specific recurrence-free survival by stages.

In the disease-specific analysis, in which distant metastasis, local recurrence, and deaths from causes unrelated to PDAC were censored and only remnant pancreatic recurrence was treated as the event, the Kaplan-Meier curves for stages 0, I, and II largely overlapped, although the overall difference was statistically significant (log-rank test, $P = 0.044$).
